# Genome-Wide Identification and Characterization of the Trehalose-6-Phosphate Synthetase Gene Family in Chinese Cabbage (*Brassica rapa*) and *Plasmodiophora brassicae* during Their Interaction

**DOI:** 10.3390/ijms24020929

**Published:** 2023-01-04

**Authors:** Liyan Kong, Jiaxiu Liu, Wenjun Zhang, Xiaonan Li, Yuting Zhang, Xueyu Chen, Zongxiang Zhan, Zhongyun Piao

**Affiliations:** Molecular Biology of Vegetable Laboratory, College of Horticulture, Shenyang Agricultural University, Shenyang 110866, China

**Keywords:** Trehalose, TPS, clubroot, *Plasmodiophora brassicae*, *Brassica rapa*

## Abstract

Trehalose is a nonreducing disaccharide that is widely distributed in various organisms. Trehalose-6-phosphate synthase (TPS) is a critical enzyme responsible for the biosynthesis of trehalose, which serves important functions in growth and development, defense, and stress resistance. Although previous studies have found that the clubroot pathogen *Plasmodiophora brassicae* can lead to the accumulation of trehalose in infected *Arabidopsis* organs, it has been proposed that much of the accumulated trehalose is derived from the pathogen. At present, there is very little evidence to verify this view. In this study, a comprehensive analysis of the *TPS* gene family was conducted in *Brassica rapa* and *Plasmodiophora brassicae*. A total of 14 *Brassica rapa TPS* genes (*BrTPSs*) and 3 *P. brassicae TPS* genes (*PbTPSs*) were identified, and the evolutionary characteristics, functional classification, and expression patterns were analyzed. Fourteen *BrTPS* genes were classified into two distinct classes according to phylogeny and gene structure. Three *PbTPSs* showed no significant differences in gene structure and protein conserved motifs. However, evolutionary analysis showed that the *PbTPS2* gene failed to cluster with *PbTPS1* and *PbTPS3*. Furthermore, cis-acting elements related to growth and development, defense and stress responsiveness, and hormone responsiveness were predicted in the promoter region of the *BrTPS* genes. Expression analysis of most *BrTPS* genes at five stages after *P. brassicae* interaction found no significant induction. Instead, the expression of the *PbTPS* genes of *P. brassicae* was upregulated, which was consistent with the period of trehalose accumulation. This study deepens our understanding of the function and evolution of *BrTPSs* and *PbTPSs*. Simultaneously, clarifying the biosynthesis of trehalose in the interaction between *Brassica rapa* and *P. brassicae* is also of great significance.

## 1. Introduction

Trehalose is a nonreducing disaccharide with two glucose molecules linked through an α, α-1,1-glucosidic bond, which has been found to exist in bacteria, fungi, algae, invertebrates, and plants [1]. Previous studies have found that trehalose is known as a living substance that exists in all living organisms and is involved in growth and development [2,3]. Trehalose biosynthesis pathways are widely distributed in nature. There are five pathways for trehalose biosynthesis that have been identified according to the different catalytic enzymes [4] (Figure S1). The first pathway is called the TS pathway; trehalose synthase (TreS) in *Pimelobacter* sp. catalyzes the conversion of maltose into trehalose by intramolecular transglycosylation [5] (Figure S1A). The second pathway (TreY/TreZ pathway) has been reported in thermophilic archaea of the genus *Sulfolobus*, which converts maltodextrin to trehalose via the catalysis of maltooligosyl trehalose synthase (TreY) and maltooligosyl trehalose trehalohydrolase (TreZ) [6] (Figure S1B). In the third pathway (TreP pathway), mainly existing in some fungi, trehalose phosphorylase (TreP) bidirectionally catalyzes the hydrolysis of trehalose in the presence of inorganic phosphorus to produce glucose-1-phosphate and a molecule of glucose. However, since this reversible reaction can only be observed in in vitro experiments, it is uncertain whether the TreP enzyme is involved in the synthesis or degradation of trehalose in vivo [7,8] (Figure S1C). The fourth pathway was found in the hyperthermophilic archaeon *Thermococcus litoralis,* involving trehalose glycosyl-transferring synthase (TreT), which is an enzyme forming trehalose and ADP from ADP–glucose and glucose [9] (Figure S1D). The above four pathways of trehalose biosynthesis have been found in several prokaryotes and archaea, but the fifth pathway (TPS/TPP pathway) is the most widespread, has been found in all prokaryotic and eukaryotic organisms that synthesize trehalose, and is the only pathway found in plants. In this pathway, the enzymatic reaction is catalyzed by trehalose 6-phosphate synthase (TPS) and trehalose 6-phosphate phosphatase (TPP), and TPS catalyzes UDP-glucose (UDPG) and glucose 6-phosphate (G6P), producing trehalose 6-phosphate (T6P) and UDP. T6P is then further catalyzed to synthesize trehalose by TPP [10,11] (Figure S1E). The trehalose synthase gene was first reported from *Pimelobacter* sp. R48 by screening the genomic DNA library [12], and later, trehalose synthase genes were identified in bacteria, fungi, plants, arthropods, and insects [10,13,14,15].

TPS performs the first step in the biosynthetic pathway of T6P and trehalose. TPS is thus of particular interest as it directly affects the concentration of both T6P and trehalose, which play vital roles in trehalose metabolism and stress resistance in plants [16]. The *Escherichia coli otsA* and *otsB* genes were identified and cloned in 1992, and *otsA* and *otsB* mutations block osmoregulatory trehalose synthesis in *E. coli* [17]. In *Saccharomyces cerevisiae*, *TPS* and *TPP* are part of the trehalose synthase complex, and the TPS homolog, TPS1, forms a complex with the TPP homolog, TPS2, and two regulatory subunits, TPS3 and TSL (trehalose synthase long chain) [18]. In many insects, multiple *TPS* genes that encode proteins harboring *TPS/OtsA*- and *TPP/OtsB*-conserved domains have been found and cloned, such as those in *Drosophila* [19], *Helicoverpa armigera* [20], and *Spodoptera exigua* [21]. Early accounts of trehalose in plants were restricted to resurrection plants, for example, *Selaginella* species and *Myrothamnus flabellifolia* [22,23,24]. Soon after, the model plant *Arabidopsis* was shown to synthesize small amounts of trehalose, and functional genes encoding *TPS* and *TPP* were found in *Arabidopsis thaliana*. The *TPS* gene family in *Arabidopsis* and rice consists of 11 members [25,26], and TPS possesses two domains, TPS (Glyco_transf_20) and TPP (Trehalose_PPase), that correspond to *OtsA* and *OtsB* genes responsible for TPS and TPP activity, respectively, in *E. coli* [27,28]. Mutants of the *Arabidopsis tps1* gene cause embryonic lethality, and *AtTPS5* functions as a negative regulator of ABA signaling and is involved in altering the trehalose content [29], indicating that trehalose synthesis plays a crucial role in plants [30].

Trehalose plays a wide range of functional roles in organisms, which is also a typical microbial sugar accumulating in multifarious symbiotic or pathogenic interactions of microorganisms with plants [31]. In some microorganisms and invertebrate animals, trehalose serves as a carbon source or an osmoprotectant [31]. In prokaryotes, trehalose frequently serves as a source of reserve energy, a compatible solute to contend with osmotic stress in spores and resting cells, and as part of the cell wall structure [32]. In insects, hemolymph trehalose is a major store of carbohydrates and an important substrate during flight [33]. In plants, the disaccharide sucrose plays a similar role as trehalose [10]. However, trehalose has been found in small amounts in only a few plants, namely specialized resurrection species, such as *Selaginella lepidophylla*, and it plays a role in stress protection, especially drought [34]. In *Arabidopsis thaliana* and other drought-resistant species, despite the existence of multiple genes encoding trehalose synthesis, only a small amount of trehalose was detected, which may be related to the co-regulation of its precursor, T6P, in the regulation of plant stress [3,35,36]. In fungi, trehalose is a reserve carbohydrate and protector against stress challenges [37]. Trehalose plays a role in the colonization of plants by pathogens [4]. Multi-host pathogen *Pseudomonas aeruginosa* synthesizes trehalose, which is required during infection of *Arabidopsis* leaves [38]. The inhibition of the biosynthesis of trehalose by the plant pathogen *Ralstonia solanacearum* contributes to the reduction of its pathogenicity, and it also indicates the important role of trehalose in the interaction between plants and pathogens [39].

Clubroot, a devastating disease affecting *Brassica* plants, is caused by the obligate biotroph protist *Plasmodiophora brassicae* and is characterized by the development of large galls on infected roots, inhibiting the uptake of nutrients and water from the soil [40]. Altered carbohydrate metabolism, including that of starch, soluble sugars, and inositol, is an important symptom of clubroot disease. Notably, a previous study found that high trehalose levels accumulated in infected tissues after *P. brassicae* inoculation [41], and this accumulation pattern was consistent with the expression of *PbTPS1*, a putative trehalose-6-phosphate synthase gene from *P. brassicae*. Scholars have speculated that a large amount of trehalose is most likely synthesized by *P. brassicae* rather than by the host. The release of trehalose synthesized by *P. brassicae* into plants might interfere with the plant’s trehalose-sensing system and alter the host’s carbohydrates in the pathogen’s favor [41]. *Plasmodiophora brassicae* is an obligate parasite that parasitizes the roots of cruciferous plants, but research on it is limited because it cannot be isolated and cultured in vitro. Therefore, it is unclear whether accumulated trehalose is synthesized by host plants or *P. brassicae*. To address this, we focused on the role of *TPS* genes in the trehalose synthesis pathway in the interaction between *Brassica rapa* and *P. brassicae* and performed genome-wide identification, characterization, and expression analysis of the *TPS* genes from *P. brassicae* and its host plant *B. rapa*.

We identified the *TPS* genes in the *B. rapa* and *P. brassicae* genomic data and investigated the functional classification, evolutionary characterization, and expression patterns of the *TPS* gene family. The present study enhances our understanding of the function and evolution of *BrTPSs* and *PbTPSs*. The study is also of great significance in clarifying the biosynthesis of trehalose in the interaction between *Brassica rapa* and *P. brassicae*.

## 2. Results

### 2.1. Trehalose Sugar Content in Cabbage Roots after Infection with P. brassicae

**Figure 1:**
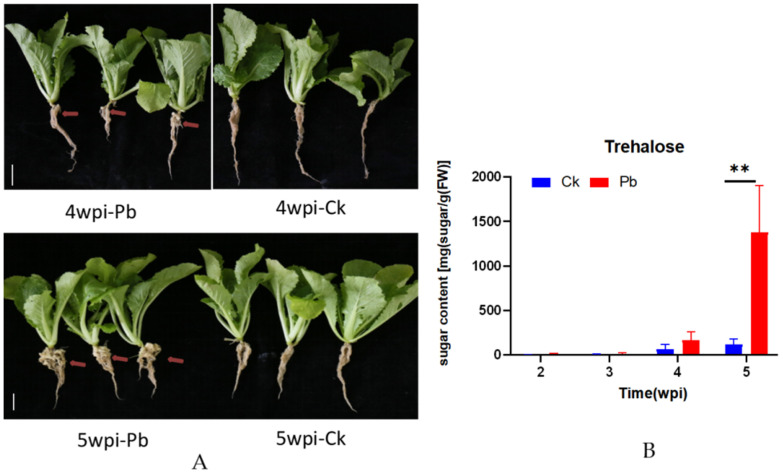
Phenotypic investigation and trehalose contents in the roots of Chinese cabbage after *plasmodiophora brassicae* inoculation. CK: inoculation-distilled water; Pb: inoculation with *P. brassicae.* (**A**) Investigation of clubroot disease of roots at 4 and the 5 weeks post-inoculation (wpi) with *P. brassicae.* The red arrow points to the location of the clubroot disease. The white scale range represents 2 cm. (**B**) The trehalose contents in the roots of Chinese cabbage after *P. brassicae* inoculation. The abscissa represents the time post-inoculation, and the ordinate represents the value of sugar content. The data represent mean values ± SDs. The asterisks indicate *p*-values (** *p* < 0.01) according to Student’s *t* test. Horizontal axis is the time point from 2 weeks to 5 weeks post-inoculation (wpi).

Five weeks after inoculation with *P. brassicae*, each of the infected Chinese cabbage roots clearly showed typical symptoms of clubroot, and from 4 to 5 weeks post-inoculation (wpi), the clubroot became increasingly obvious (Figure 1A). To investigate changes during the development of clubroot, the trehalose content of the roots was analyzed at four different stages after infection (from the second week to the fifth week) (Figure 1B). From 2 to 3 wpi, the trehalose content was extremely low, and there was no difference between the healthy and infected plants. However, trehalose accumulated at 4 wpi when the trehalose content was slightly higher in infected plants than in healthy plants. Most notably at 5 wpi, trehalose increased greatly in infected plants, with a 1000-fold increase relative to healthy plants. This indicates that *P. brassicae* infection leads to the accumulation of a large amount of trehalose in Chinese cabbage roots.

### 2.2. Identification of TPS Family Members in B. rapa and P. brassicae

To analyze the biosynthesis of trehalose, we identified *TPS* genes in the *B. rapa* and *P. brassicae* genomes. Based on similarities with the 11 *Arabidopsis AtTPSs*, a total of 14 *BrTPSs* were identified in the *B. rapa* genome and named *BrTPS1a* to *BrTPS11* based on their identity with *AtTPSs.* Among the 14 *BrTPSs*, 8 *BrTPS* genes had two copies corresponding to *AtTPSs*, while the orthologous gene of *AtTPS3* was not found in the *B. rapa* genome. The majority of the *BrTPS* gene-coding sequences were about 2700 base pairs (bp), and the length of amino acid residues was about 900 aa. The shortest coding sequence of *BrTPS5a* was only 390 bp, and the length of the amino acids was 129 aa. The isoelectric point (pI) value of *BrTPSs* ranged from 4.7 to 9.48, and the protein molecular weight ranged from 14.7 to 165.8 kDa. Subcellular localization predictions showed that they were mainly localized in the chloroplast, vacuole, and cytoplasm (Table 1).

A total of three *TPS* genes were acquired by keyword search against the *P. brassicae* genome in NCBI’s nr database, namely *PbTPS1*, *PbTPS2*, and *PbTPS3*. The coding sequence length of the three *PbTPS* genes was longer than 2500 bp, and the length of the amino acid ranged from 853 to 860 aa. The molecular weight ranged from 95.50 to 96.49 kDa, and the predicted pI value ranged from 6.18 to 6.93. Subcellular localization prediction indicated that the three *PbTPS* proteins were located in the cytoplasm (Table 2).

### 2.3. Phylogenetic Analysis of BrTPSs and PbTPSs

**Figure 2:**
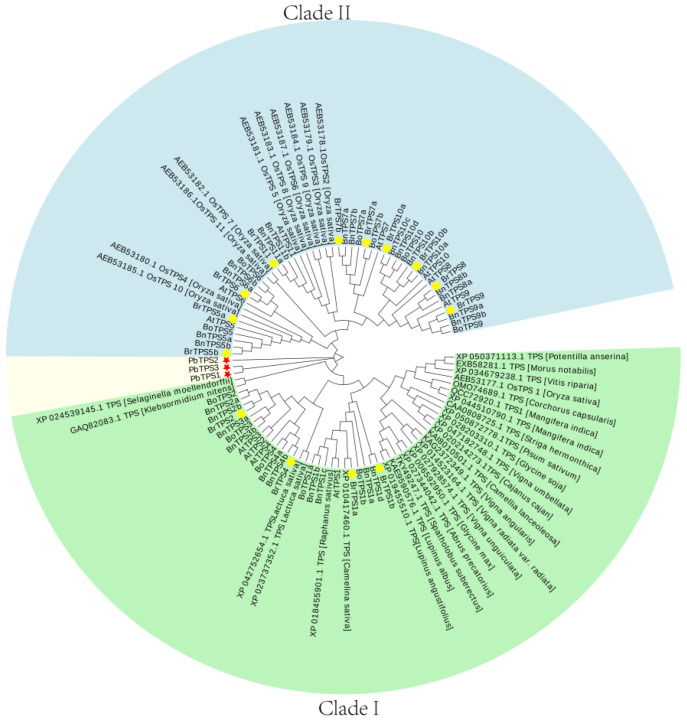
Phylogenetic relationship of Br*TPS* and *PbTPS*. All TPS proteins were divided into three subgroups, represented by three colors. The green color represents Class I proteins, the blue color represents Class II proteins. The yellow circle symbol represents the BrTPS proteins. The unrooted phylogenetic tree was constructed using the neighbor-joining (NJ) method with 1000 bootstrap replications by MEGA6 software.

To analyze the evolutionary relationships of *BrTPS* and *PbTPS* genes in *B. rapa* and *P. brassicae*, an unrooted phylogenetic tree was constructed using full-length amino acid sequences (Figure 2). In total, The TPS sequences from 30 species were assessed in the phylogenetic tree (Supplementary Table S2). The *TPSs* of *B. rapa* were grouped into two major clades; *BrTPS1a* to *BrTPS4* belonged to Clade I, and *BrTPS5a* to *BrTPS11* belonged to Clade II. The *PbTPSs* belonged to a separate clade, which indicated that PbTPS is far away from other species in evolution. In addition, *PbTPS3* and *PbTPS1* were clustered into the same branch; *PbTPS2* failed to cluster with them, which indicated that there are sequence differences between *PbTPSs*, and there may be functional differences.

### 2.4. Gene Structure and Conserved Domain Analyses of BrTPSs and PbTPSs

**Figure 3:**
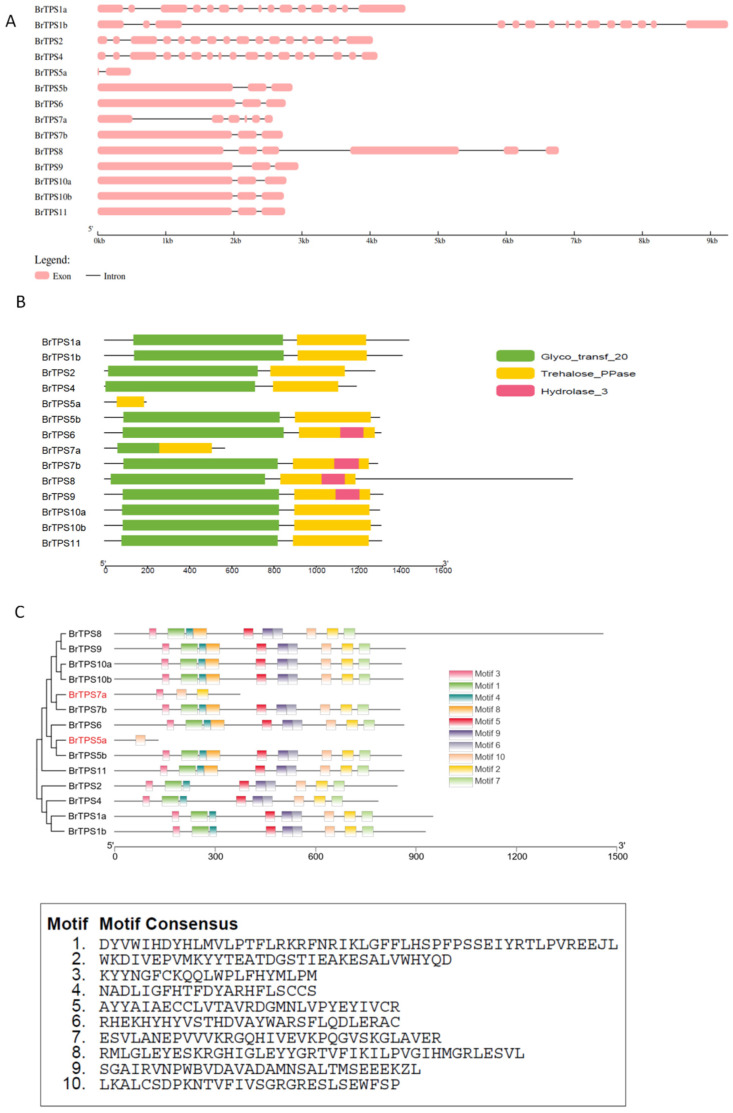
Gene structures, domains, and motifs of the BrTPS family. (**A**) Exon/intron organization of *BrTPS* genes. Pink boxes represent exons and black lines of same length represent introns. The length of exons can be inferred by the scale at the bottom. (**B**) The conserved domain analysis of BrTPS protein. Trehalose-6-phosphate synthase (TPS) domain (Glyco_transf_20), trehalose-6-phosphate phosphatase (TPP) domain (Trehalose_PPase), and haloacid dehalogenase-like hydrolase domain-containing 3 (Hydrolase 3) are shown by green, yellow, and red, respectively. (**C**) Conserved motifs of BrTPS proteins. Ten putative motifs are indicated in different colored boxes. The details of the motifs are listed at the bottom.

**Figure 4:**
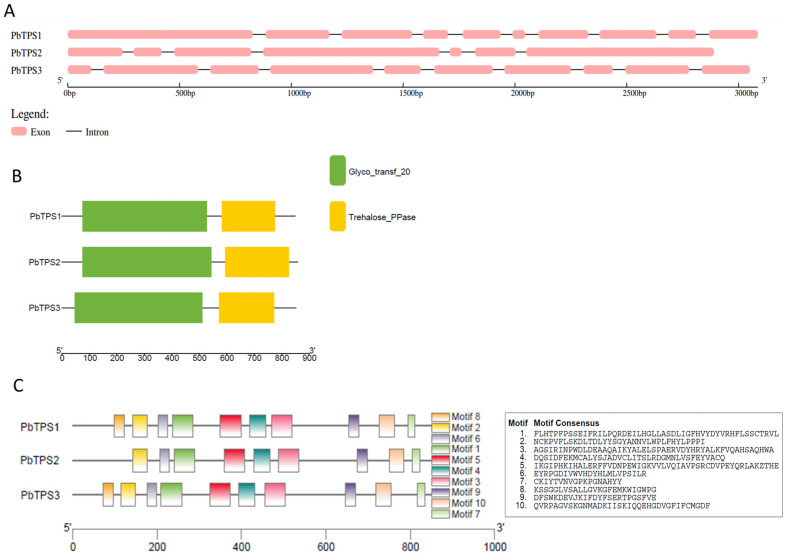
Gene structures, domains, and motifs of the PbTPS family. (**A**) Exon/intron organization of *PbTPS* genes. Pink boxes represent exons and black lines with same length represent introns. The length of exons can be inferred by the scale at the bottom. (**B**) The conserved domain analysis of PbTPS protein. Trehalose-6-phosphate synthase (TPS) domain (Glyco_transf_20), trehalose-6-phosphate phosphatase (TPP) domain (Trehalose_PPase) are shown by green and yellow, respectively. (**C**) Conserved motifs of PbTPS. Ten putative motifs are indicated in different colored boxes. The details of the motifs are listed on the right.

Gene structure analysis is an available method for understanding gene evolutions and their potential roles. Thus, the structures of the *BrTPS* and *PbTPS* genes were investigated. For 14 *BrTPS* genes, the number of exons ranged from 2 to 17, and the majority of *BrTPS* genes (accounting for 42.8%) had three exons. *BrTPS4* contained the most exons (17), whereas *BrTPS*5a harbored the fewest exons (2). Interestingly, *BrTPS* genes clustered in the same clade generally possessed a similar exon–intron structure, and genes in Clade I had more exons than genes in Clade II (Figure 3A). Generally, analysis of these *TPS* gene structures showed that the conserved exon–intron structure within each cluster agreed with the classification of *TPS* genes in an NJ phylogenetic tree based on TPS sequences (Figure 2). For *PbTPS* genes, the number of exons ranged from 7 to 10. Among them, *PbTPS1* and *PbTPS3* had 10 exons, but *PbTPS2* contained 7 exons (Figure 4A).

Domain analysis of the identified BrTPS and PbTPS protein sequences showed that, except BrTPS5a, the other TPS proteins contained a TPS structure domain (Glyco_transf_20) located at the N-terminal and a TPP domain (Trehalose_PPase) at the C-terminal. However, BrTPS5a contained only the TPP domain (Figure 3B and Figure 4B). To further elucidate the structural and functional features of BrTPSs and PbTPSs, 10 conserved motifs of the TPS proteins were identified using the MEME program. For BrTPS proteins, these motifs were conserved in most BrTPS proteins, except BrTPS7a and BrTPS5a, which harbored one and three motifs, respectively. The lengths of these motifs ranged from 21 to 50 amino acids. Among them, Motifs 1, 3, 4, 5, 6, 8, and 9 together composed the TPS domain (Glyco_transf_20). Motifs 2, 7, and 10 composed the TPP domain (Trehalose_PPase) (Figure 3C). For PbTPS proteins, these motifs were almost conserved in PbTPS proteins, except PbTPS2, which lacked Motifs 5 and 8. The lengths of these motifs ranged from 18 to 50 aa. According to Figure 4C, Motifs 1, 2, 3, 4, 5, 6, and 8 were located in the TPS domain, and Motifs 7, 8, and 10 were located in the TPP domain.

### 2.5. Chromosomal Location of BrTPS Genes

**Figure 5:**
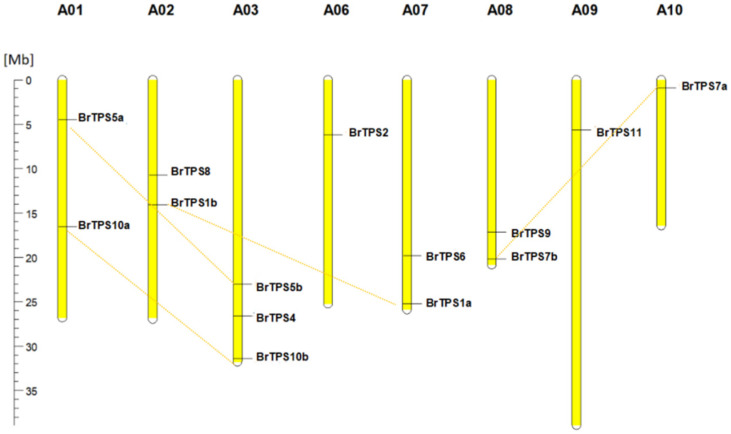
The *TPS* gene locations in *B. rapa* chromosomes. The chromosomes are represented by yellow bars. The chromosomal position of each *BrTPS* gene was mapped according to the *B. rapa* genome. The chromosome number is indicated at the top of each chromosome. The scale is in megabases (Mb).

To analyze the distribution of *BrTPS* genes in the genome, we showed their position on each chromosome based on the *B. rapa* genome database (Figure 5). Fourteen *BrTPS* genes were dispersed on eight chromosomes. Each chromosome contained 1–3 *BrTPS* genes. Chromosome A03 contained three *BrTPS* genes (*BrTPS4*, *BrTPS5b*, and *BrTPS10b*), and chromosomes A06, A09, and A10, each contained one *BrTPS* gene (*BrTPS2*, *BrTPS11*, and *BrTPS7a*, respectively), while chromosomes A01, A02, A07, and A08 each had two *BrTPS* genes. A duplication event was identified in the *B. rapa* genomes. Four *AtTPS* genes (*AtTPS1*, *-5*, *-7*, and *-10*) were duplicated in *B. rapa*, and the *BrTPS* paralogous genes were dispersed on different chromosomes.

### 2.6. Identification of Cis-Acting Elements in the Promoter Region of BrTPS Genes

**Figure 6:**
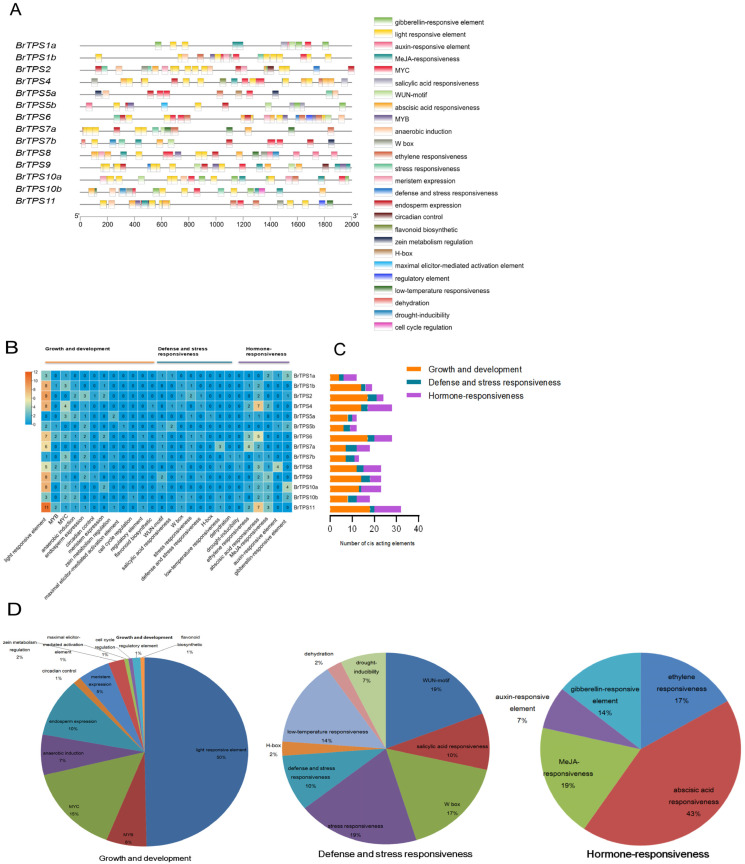
Predicted cis-elements in *BrTPS* gene promoters. Promoter sequences (2000 bp) of 14 *BrTPS* genes were analyzed using the PlantCARE database. (**A**) Kind and position of cis-acting elements in *BrTPSs*. (**B**) numbers of cis-acting elements. The gradient blue colors and numbers in the grid indicate the number of different cis-acting elements in *BrTPSs*. (**C**) Number of cis-acting elements in 14 *BrTPS* genes containing three category; histograms with different colors represent different categories. (**D**) Proportion of different cis-acting elements in each category.

To ascertain the potential biological roles of *BrTPS* genes in *B. rapa*, 2000 bp sequences upstream of the start site of *BrTPS* genes were used to identify the potential cis-acting elements in the promoter region. A total of 285 functionally annotated cis-acting elements were predicted in these genes. Many cis-acting elements were involved in light responsiveness, stress responsiveness, hormone responsiveness, site binding, and other functions (Figure 6A). Generally, the cis-acting elements were roughly classified into three categories of cis-elements linked to growth and development, defense and stress responsiveness, and hormone responsiveness (Figure 6B). The cis-acting elements in growth and development were the most involved, followed by the cis-acting elements in response to hormones, and the elements in defense and stress were the least involved. Among the 14 *BrTPS* genes, *BrTPS1a* had the least cis-acting elements, while *BrTPS11* contained the most (Figure 6C). In the plant growth and development category (159/285), 79 cis-elements (accounting for 50%) were involved in light responsiveness, which accounted for the largest proportion in this category, and this element was contained in all 14 *BrTPS* genes. Eleven cis-elements were involved in anaerobic induction, 16 in endosperm expression, 8 in meristem expression, 4 in zein metabolism regulation, 10 and 24 as MYB and MYC binding sites, respectively, and 2 in circadian control and regulation. Some *BrTPS* genes elicit specific cis-acting elements, such as the maximal elicitor-mediated activation element only in *BrTPS5b*, the cell cycle regulatory element only in *BrTPS10b*, and the flavonoid biosynthetic element only in *BrTPS4*, which indicates the specificity of the function of these genes (Figure 6B,D). In the defense and stress responsiveness category (42/285), eight cis-elements (accounting for 19%) were involved as WUN-motifs, eight (accounting for 19%) in stress responsiveness, six (accounting for 14%) in low-temperature responsiveness, four (accounting for 10%) in salicylic acid responsiveness, four in defense and stress responsiveness, and three (accounting for 7%) in drought inducibility. Additionally, the W-box element (accounting for 17%) was found in seven *BrTPS* genes. The dehydration responsiveness element (accounting for 2%) was found in *BrTPS7b* (Figure 6B,D). In the hormone responsiveness category (84/285), various cis-elements were related to ethylene responsiveness (accounting for 17%), abscisic acid responsiveness (accounting for 43%), MeJA responsiveness (accounting for 19%), auxin responsiveness (accounting for 7%), and gibberellin responsiveness (accounting for 14%). Notably, the largest number of cis-elements was in the abscisic acid-responsive elements. The results suggest that most *BrTPS* genes might be acid-induced and/or -repressed genes (Figure 6D).

### 2.7. Expression of BrTPS Genes under P. brassicae Infection

**Figure 7:**
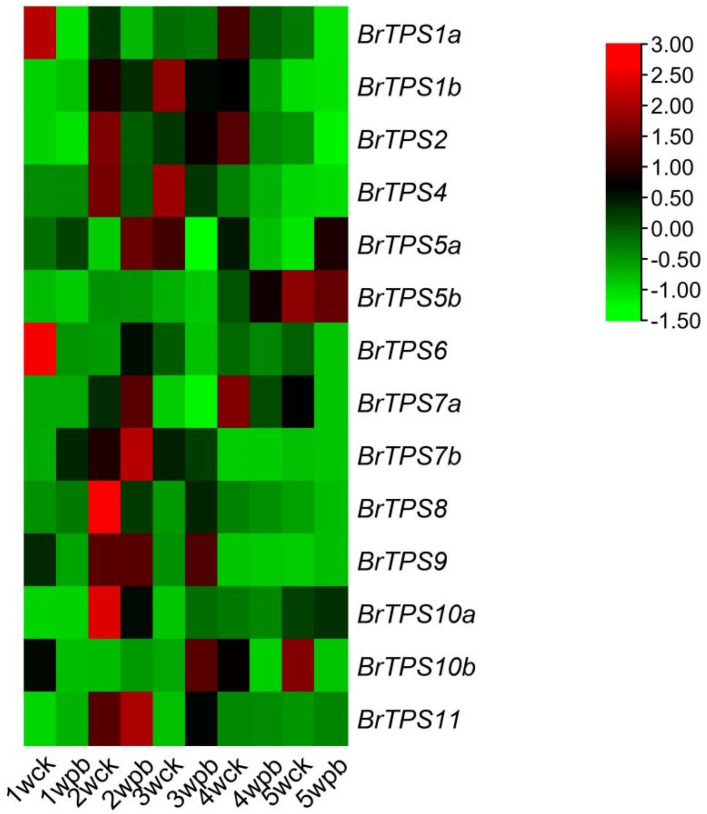
Relative Expression profiles of *BrTPS* genes in the Chinese cabbage root after of *P. brassicae*. infection. CK represents inoculation with water; Pb represents inoculation with *P. brassicae*. Expression of each *BrTPS* gene was measured by qRT-PCR from 1 week to 5 weeks post-inoculation. The expression levels of genes are presented using fold change values transformed to Log2 format. The data indicate the relative expression levels normalized to that of the internal control *18srRNA*. Red and green colors correspond to up- and downregulations of the BrTPS gene expressions, respectively.

To further explore the role of *BrTPS* genes responsive to *P. brassicae* infection, the expression patterns of 14 *BrTPSs* were determined in the roots of Chinese cabbage from 1 to 5 wpi using qRT-PCR (Figure 7). Generally, *BrTPS* genes exhibited distinct time-specific expression profiles, suggesting the functional divergence of *BrTPS* genes at different stages during growth and development. We found that most of the genes were highly expressed at 1 to 3 wpi but downregulated at 4 to 5 wpi by investigating the differences in the expression of these 14 *BrTPS* at five stages after infection with *P. brassicae* between the Ck and Pb plants. At 1 wpi, the expression levels of four genes (*BrTPS1b*, *BrTPS5a*, *BrTPS7b*, and *BrTPS11*) in Pb plants were significantly higher than those in Ck plants; at 2 wpi, the expression levels of six genes (*BrTPS5a*, *BrTPS6*, *BrTPS7a*, *BrTPs7b*, *BrTPs10b*, and *BrTPS11*) in Pb plants were significantly higher than those in Ck plants; at 3 wpi, the expression levels of six genes (*BrTPS2*, *BrTPS8*, *BrTPS9*, *BrTPs10a*, *BrTPs10b*, and *BrTPS11*) in Pb plants were upregulated than those in Ck plants. Interestingly, only *BrTPS5b* and *BrTPS5a* were upregulated at 4 and 5 wpi, compared to Ck plants. Since trehalose was mainly accumulated at 4 to 5 wpi, it was worth noting that although *BrTPS5a* was upregulated relative to CK plants at 5 wpi, it has a higher expression at 2 wpi, and trehalose did not accumulate in a large amount at that time; similarly, we also noticed that the expression of *BrTPS5b* in Ck plants was higher than that in Pb plants at 5 wpi. Therefore, we speculated that *BrTPS* genes may play little role in trehalose synthesis.

### 2.8. Expression Analyses of PbTPS Genes

**Figure 8:**
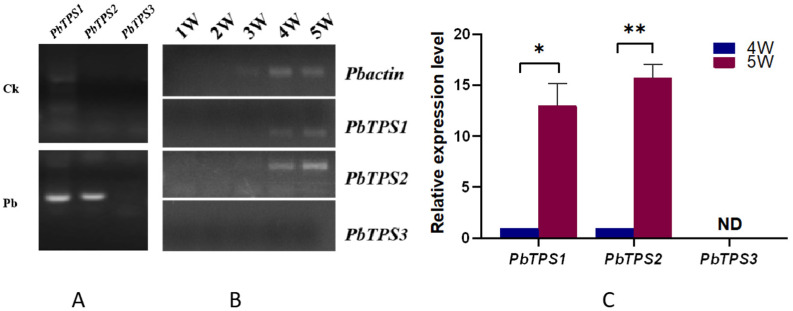
Analysis *PbTPS* genes in the Chinese cabbage root after *P. brassicae* infection. (**A**) Reverse-transcription polymerase chain reaction analysis of the expression of *PbTPS* in infected and healthy plants roots, the template of PCR reaction was root cDNA mixture from 1 week to 5 weeks post-inoculation (wpi). CK: heathy plants; Pb: infected plants. (**B**) Real-time PCR analysis of the expression of *PbTPSs* in infected plants roots. 1W–5W represent five time points from 1 week to 5 weeks post-inoculation with *P. brassicae.* (**C**) qRT-PCR analysis of the expression of *PbTPSs* in infected plants roots at 4 wpi and 5 wpi. Values represent the mean and standard deviation of triplicate results. “ND” means not detected. Asterisks indicate values that are statistically significantly different from the 4 wpi using Student’s *t* test. (* *p* < 0.05; ** *p* < 0.01).

To determine the role of *PbTPS* genes correlated with the accumulation of trehalose, a semi-quantitative PCR analysis was carried out in the root samples of Pb and Ck plants to analyze the expression of the three *PbTPS* genes. *PbTPS* genes were amplified only in Pb plants (Figure 8A). A semi-quantitative PCR analysis of *PbTPSs* was carried out at the five stages (from 1 to 5 wpi) after inoculation. The transcripts were detected in the fourth and fifth weeks after inoculation, while the *PbTPS3* gene was not detected at any stage (Figure 8B). Quantitative RT-PCR showed that *PbTPS1* and *PbTPS2* were significantly upregulated from 4 to 5 wpi. Specifically, *PbTPS2* had the highest expression level, which was increased by more than 10-fold (Figure 8C).

## 3. Discussion

Plant pathogens tend to alter carbohydrate transport and distribution in host tissues, a process involving different types of sugars [42,43]. It has been proposed that the pathogen attempts to manipulate the carbohydrate metabolism of the host in the pathogen’s favor [44]. Previous studies have shown the accumulation of soluble sugars in Chinese cabbage tissues after *P. brassicae* infection, suggesting that *P. brassicae* infection could trigger active sugar translocation between the sugar-producing tissues and the clubbed tissue [45]. In addition, a previous study reported a significant accumulation of trehalose in *Arabidopsis* roots and hypocotyls after *P. brassicae* infection. In this study, we observed that the contents of trehalose were significantly increased in Chinese cabbage clubroots at 5 wpi, a 1000-fold increase compared to control plants. Almost no trehalose was detected in infected plants at 2 and 3 wpi, indicating that trehalose was mainly synthesized during the later stages of clubroot development. Simultaneously, smaller amounts of trehalose were also found in healthy plants, indicating that plants are capable of synthesizing small amounts of trehalose during growth and development. This result was consistent with previous reports that trehalose accumulates in the roots and hypocotyls of infected *Arabidopsis* [41].

TPS is the primary enzyme responsible for catalyzing the trehalose formation; therefore, elucidating the role of *TPS* genes in trehalose biosynthesis and their identification and analysis in both host plants and *P. brassicae* are of interest. Genes encoding TPS have been identified in many plants in the form of a gene family [46]. The *Arabidopsis TPS* gene family contains 11 members (*AtTPS1–11*) [16], rice contains 11 members (*OsTPS1–11*) [26], tomato contains 10 members (*SlTPS1–10*) [47], and watermelon contains 7 members (*ClTPS1–7)* [3]. However, the *TPS* gene family in *B. rapa* and *P. brassicae* has not been well studied. Herein, 14 *BrTPS* genes were identified in the *B. rapa* genome, and 3 *PbTPS* genes were identified in the *P. brassicae* genome. According to their gene structure and enzyme activity, the *TPS* family of genes in plants are classified into two major clades: Clades I and II. In the *Arabidopsis* genome, four genes belong to Clade I (*AtTPS1–4*) and seven to Clade II (*AtTPS5–11*) [25]. *Brassica rapa TPS* genes were also divided into two subfamilies: Clades I (*BrTPS1a* to *BrTPS4*) and II (*BrTPS5a* to *BrTPS11*), which was consistent with the classification in *Arabidopsis* [25] and rice [26]. There were 14 *BrTPS* genes, which was greater than the number of genes in *A. thaliana* and rice. In addition, *Arabidopsis*, a species closely related to the *Brassica* genus, contained more *TPS* genes than *B. rapa*, suggesting that there may be functional redundancy or divarication between the TPS members. However, the occurrence of gene loss during polyploid speciation was also found in the *B. rapa* genome corresponding to *Arabidopsis TPS* genes. For example, the *AtTPS3* homolog was absent in the *B. rapa* genome. Some traits differed between the two *BrTPS* clades, such as in gene structure and gene length, but especially the gene structure. We observed that the structure of the *BrTPS* genes in Clades I and II was very different, and the number of introns and exons in Clade I was significantly greater than that in Clade II. Previous studies have suggested three mechanisms (exon/intron gain/loss, exonization/pseudoexonization, and insertion/deletion) that may lead to differences in gene structure [48], and a close relationship between the structure and function of genes has been observed [49]. Moreover, it was stated that the exon/intron number could affect the expression level; genes with fewer introns might be quickly induced [50,51]. Therefore, the *BrTPS* genes in Clades I and II may have experienced functional differentiation during evolution. Domain analysis showed that most BrTPS proteins had a TPS domain (Glyco_transf_20) at the N-terminus and a TPP domain (Trehalose_PPase) at the C-terminus, which was consistent with the results of other studies [11,16,27,46]. However, BrTPS5a contains only a Trehalose_PPase domain. Similarly, three GhTPS proteins (GhTPS6, GhTPS4, and GhTPS9) in cotton also lack a TPP domain [11], and the loss of the domain may be the result of evolution [46]. Conserved motif analysis demonstrated that the conserved number of BrTPS5a and BrTPS7a was less than that of the other BrTPSs, which may be related to the length of the genes. As for PbTPSs, there was no significant difference in the gene structure and conserved motif of the three PbTPSs, and all possessed a TPS domain at the N-terminal and a TPP domain at the C-terminal. Cis-acting elements were involved in the regulation of gene expression [46]. Previous studies have shown that *TPS* genes provide stress tolerance in different plant species. For example, plants overexpressing *AtTPS1* improved drought resistance in *Arabidopsis* [52]. Overexpression of *OsTPS8* was adequate to confer enhanced salinity tolerance [53], and watermelon *ClTPS3* overexpression in *Arabidopsis thaliana* significantly improved salt tolerance [3]. In this study, a variety of signal response elements were contained in the promoter regions of *BrTPS* genes. Moreover, many *BrTPS* genes contain different stress response elements, indicating that *BrTPS* genes are involved in stress defense processes.

Trehalose is not only used as a stored energy but also serve as a protectant, when encountering drought, cold, osmotic stress, oxidation, and other stress conditions [5]. To determine whether large amounts of trehalose were synthesized by the host or *P. brassicae*, we examined the expression patterns of *BrTPS* and *PbTPS* genes under *P. brassicae* stress. At 4 and 5 wpi, the expression level of most *BrTPS* genes in Pb plants was not significantly higher than that in Ck plants, except for *BrTPS5a* and *BrTPS5b*. Although the expression level of *BrTPS5a* in Pb plants was higher than that in Ck at 5 wpi, its expression level was still lower than that at 2 wpi, in which trehalose did not accumulate. Therefore, the substantial accumulation of trehalose is likely not due to synthesis within the host plant. According to a previous study, the resting spores of *P. brassicae* were detected to contain 14.7 mg/g dry weight of trehalose, which was much higher than that of the infected *Arabidopsis* tissue, which indicated that *P. brassicae* is capable of synthesizing a large amount of trehalose. This result correlated with the expression of *PbTPS1* in resting spores [41]. Here, we identified other *TPS* genes in *P. brassicae* and analyzed *PbTPS* gene expression patterns, showing that *PbTPS3* was not expressed and that *PbTPS1* and *PbTPS2* were significantly upregulated from 4 to 5 wpi. Our results are consistent with the previous study and further confirm the view that increased trehalose is probably synthesized by pathogens rather than by the host plant. Most fungi and bacteria produce trehalose, and the virulence of some of these plant pathogens is dependent on their trehalose metabolism [10,38,54]. In addition, trehalose plays a key role in protecting bacteria and fungi against a range of stressors [55] and is abundant in the spores of fungi and yeast. For example, more than 7% trehalose and trace amounts of glucose were found on a dry-weight basis in the spores and macrocysts of *Dictyostelium mucoroides* but not in other lifecycle stages; thus, trehalose restricted in the spores and macrocysts was utilized as energy for germination [56]. Furthermore, trehalose in *Neurospora tetrasperma* served to activate the ascospores [57]. Similarly, trehalose in *P. brassicae* may be used as a stress protectant to protect the resting spores of *P. brassicae* from various stressors. The resting spores of *P. brassicae* have been reported to survive in the soil for decades [58], perhaps because excess energy allows the germination of resting spores in *P. brassicae*.

In summary, a large amount of trehalose accumulated in the roots of *B. rapa* during clubroot development. We identified 14 *BrTPS* genes from *B. rapa* and 3 *PbTPS* genes from *P. brassicae*. The phylogenetic analysis and gene structure, conserved motif, and cis-acting element analyses of *BrTPS* genes were performed. Expression analysis showed that most *BrTPS* genes were not significantly induced by *P. brassicae* infection, but the expression of the three *PbTPS* genes of *P. brassicae* was upregulated. Therefore, much of the accumulated trehalose most likely originated from the pathogen, and the trehalose in the resting spores of *P. brassicae* may serve as energy for germination.

## 4. Materials and Methods

### 4.1. Plant Material and P. brassicae Inoculation

The Chinese cabbage susceptible variety “BJN3-2” was used as a host and maintained in the culture room under a 16 h light/8 h dark photoperiod at 25 °C. Chinese cabbage plant material was sown in the greenhouse of Shenyang Agricultural University in May under natural conditions. The *P. brassicae* resting spores of a single-spore isolate (Pb4) were collected from clubbed roots and diluted to a density of 1 × 10^7^ spores/mL with sterile distilled water until inoculation. “BJN3-2” and the single-spore isolate (Pb4) of *P. brassicae* were preserved in the Laboratory of Vegetable Molecular Biology, College of Horticulture, Shenyang Agricultural University. The roots of 2-week-old cabbage seedlings were inoculated with 1 mL of a resting spore suspension, and plants inoculated with an equal volume of distilled water were used as controls. Plants were sampled and analyzed at 5 time points (from the first week to the fifth week) after inoculation. Each treatment was carried out with three biological replicates, and each replicate contained eight plants.

### 4.2. Soluble Sugar Extraction and Trehalose Content Determination

Soluble sugars were extracted from the roots of Chinese cabbage at weeks 2, 3, 4, and 5 after P. brassicae inoculation according to the previous method [45]. The GC-MS QP2010 ultra instrument (Shimadzu, Kyoto, Japan) was used for the gas quality analyses. The detailed setting information is as follows: the inlet temperature was 300 °C, the split ratio was 10:1, the carrier gas was high-purity helium, and the flow rate was 1 mL/min. The heating program was as follows: 120 °C for 3 min, 5 °C/min to 210 °C for 5 min, and 15 °C/min to 300 °C for 10 min. The ion source temperature was 200 °C, and the interface temperature was 280 °C. The solvent removal time was 3 min, and the scanning m/z was 45–500. Soluble sugars were extracted from three biological replicates at each time point. Gas quality analyses were repeated three times for each treatment.

### 4.3. Identification of TPS Genes in B. rapa and P. brassicae

To identify the *TPS* genes in the *B. rapa* genome, amino acid sequences of 11 *A. thaliana* Please use consistent spacing around headers. *TPS* (*AtTPS*) genes were selected as bait to search the *B. rapa* database (http://brassicadb.cn/#/) (accessed on 9 June 2021) by performing a BLASTP analysis. The paralogous genes of *BrTPSs* were named according to the level of sequence similarity with the corresponding genes in *AtTPSs*, and a suffix (a, b, c, etc.) was added to the gene according to the E-value from high to low. The *TPS* genes of *P. brassicae* were screened using the keywords “*Plasmodiophora brassicae* trehalose-6-phosphate synthase” from the whole genome sequencing data of *P. brassicae* in the National Center for Biotechnology Information database (NCBI) (https://www.ncbi.nlm.nih.gov/) (accessed on 5 May 2022) [59,60,61,62]. The Conserved Domains database (http://pfam.xfam.org/) (accessed on 5 May 2022) was used to ensure that all candidate TPSs contained the TPS domain. Proteins that contained typical domains (Glyco_transf_20 or Trehalose_PPase) were selected. The physicochemical parameters, including isoelectric points (pI) and molecular weight (kDa), were calculated using the online ExPASy2 database (https://www.expasy.org/resources/compute-pi-mw) (accessed on 10 May 2022). Subcellular localization prediction was performed using Cell-PLoc 2.0 (http://www.csbio.sjtu.edu.cn/bioinf/Cell-PLoc-2/) (accessed on 10 May 2022).

### 4.4. BrTPS and PbTPS Phylogenetic Evolution Analysis

The TPS protein sequences of *Arabidopsis* were downloaded from the *Arabidopsis* genome database (https://www.arabidopsis.org/index.jsp) (accessed on 9 June 2021). *Brassica napus* and *Brassica oleracea* TPS protein sequences were downloaded from the BRAD database (http://brassicadb.cn/#/) (accessed on 9 June 2021). The *TPS* genes in *Arabidopsis*, *B. napus*, *B. oleracea*, *B. rapa*, and *P. brassicae* were named *AtTPSs*, *BnTPSs*, *BoTPSs*, *BrTPSs*, and *PbTPSs*, respectively. Other organisms’ TPS protein sequences were subjected to protein BLAST in NCBI’s nr database (Non-Redundant Protein Sequence Database). All TPS protein sequences were used for phylogenetic analysis. Multiple sequence alignment of all TPS protein sequences was conducted with the ClustalW program [63]. An unrooted neighbor-joining (NJ) phylogenetic tree was constructed using MEGA6 [64] software with a bootstrap test of 1000 replicates.

### 4.5. Analysis of BrTPS and PbTPS Gene Structures and Conserved Motifs

The exon–intron structures of the *BrTPS* and *PbTPS* genes were identified using the Gene Structure Display Server (GSDS, http://gsds.cbi.-pku.edu.cn/) (accessed on 11 May 2022) [65]. The putative conserved motifs of BrTPS and PbTPS proteins were then identified by online MEME tool (http://meme-suite.org/tools/meme) (accessed on 11 May 2022). Protein sequences were analyzed using the MEME program with any number of repetitions. The maximum retrieval value for the motif was set to 10, and the other parameters were set to default. The conserved domains were visualized using the TBtools software package (http://www.tbtools.com/) (accessed on 11 May 2022) [66].

### 4.6. Chromosomal Location and Cis-Acting Element Analysis of BrTPS Genes

The chromosomal positions of the *BrTPSs* genes were acquired from the BRAD database (http://brassicadb.cn/#/) (accessed on 11 May 2022). MapChart software [67] was used to map *BrTPS* genes’ chromosomal positions and relative distances; the 2 kb sequences in the *BrTPS* genes’ upstream region were obtained using the BRAD database (http://brassicadb.cn/#/) (accessed on 12 May 2022). Subsequently, the online tool PlantCARE (http://bioinformatics.psb.ugent.be/webtools/plantcare/html/) (accessed on 12 May 2022) was employed to investigate putative cis-regulatory elements in the promoter region of *BrTPS* genes in *B. rapa*. TBtools [66] software was used to visualize these cis-regulatory elements.

### 4.7. Total RNA Extraction and Quantitative Real-Time PCR

RNA was extracted from Chinese cabbage roots using Trizol reagent (Tiangen, Beijing, China) according to the manufacturer’s protocol. First-strand cDNA was obtained using reverse transcription performed with MonScript™ RTIII Super Mix (Monad, China). Quantitative real-time RT-PCR was performed using the SYBR^®^ Green Premix Pro Taq HS qPCR Kit AG11701 (Accurate Biotechnology, Changsha, China). For reference genes, *18s* and *Pbactin* were used for *B. rapa*. and *P. brassicae*, respectively. PCR reactions were carried out in triplicate with three independent RNA samples, and the primers were synthesized by Hongxun Biological Company (Suzhou, China) and are listed in Supplementary Table S1. The 2^−ΔΔCt^ [68] method was used to analyze the relative gene expression level.

### 4.8. Statistical Analysis

The data are presented as the means ± SDs (standard deviations). Other statistical evaluations and significance tests were performed via Student’s *t*-tests with the SPSS statistical software (Version 19.0; SPSS, Inc., Chicago, IL, USA). The data were graphically analyzed using GraphPad Prism V8.0.2 (San Diego, CA, USA).

## Figures and Tables

**Table 1:** The information of *TPS* genes in *B. rapa*.

Gene Name	Gene Accession Number	*Arabidopsis homologous* Genes	CDS Length (bp)	Amino Acids Length (aa)	pI ^1^	Molecular Weight (KDa)	Predicted Subcellular Localization
*BrTPS1a*	Bra035049	AT1G78580	2853	950	6.63	106.9	Cell wall/cytoplasm/vacuole
*BrTPS1b*	Bra008366	AT1G78580	2787	928	6.87	104.5	Vacuole
*BrTPS2*	Bra026011	AT1G16980	2535	844	6.04	95.7	Chloroplast/vacuole
*BrTPS4*	Bra019043	AT4G27550	2361	786	6.11	88.6	Chloroplast/vacuole
*BrTPS5a*	Bra040180	AT4G17770	390	129	9.48	14.7	Nucleus/vacuole
*BrTPS5b*	Bra012642	AT4G17770	2574	857	5.92	96.9	Vacuole
*BrTPS6*	Bra004054	AT1G68020	2592	863	6.03	98	Cytoplasm/Vacuole
*BrTPS7a*	Bra015497	AT1G06410	1125	374	4.7	42.9	Cytoplasm/vacuole
*BrTPS7b*	Bra030651	AT1G06410	2559	852	5.54	96.9	Vacuole
*BrTPS8*	Bra007906	AT1G70290	4380	1459	6.81	165.8	Chloroplast/vacuole
*BrTPS9*	Bra016328	AT1G23870	2607	868	5.85	98.6	Vacuole
*BrTPS10a*	Bra031526	AT1G60140	2574	857	6.04	97	Chloroplast/vacuole
*BrPS10b*	Bra017888	AT1G60140	2586	861	6.01	96.9	Chloroplast/vacuole
*BrTPS11*	Bra038548	AT2G18700	2595	864	6.16	97.8	Chloroplast/vacuole

^1^ The isoelectric point of the protein.

**Table 2:** The information of *TPS* genes in *P brassicae*.

Gene Name	GenBank ID	CDS Length (bp)	Amino Acids Length (aa)	pI ^1^	Molecular Weight (KDa)	Predicted Subcellular Localization
*PbTPS1*	CAL69928.1	2853	860	6.31	96.49	Cytoplasm
*PbTPS2*	CEP00348.1	2577	858	6.93	95.87	Cell membrane/cytoplasm
*PbTPS3*	CEO95489.1	2562	853	6.18	95.50	Cytoplasm

^1^ The isoelectric point of the protein.

## Data Availability

Not applicable.

## Supplementary Materials

The following supporting information can be downloaded at: https://www.mdpi.com/article/10.3390/ijms24020929/s1.
