# Supplementary material for: Genome-Wide Identification and Characterization of the Trehalose-6-Phosphate Synthetase Gene Family in Chinese Cabbage (Brassica rapa) and Plasmodiophora brassicae during Their Interaction
Source: Int J Mol Sci. 2023 Jan 4;24(2):929. doi: 10.3390/ijms24020929 (PMC9864397; doi:10.3390/ijms24020929)
Supplement: Supplementary file 1 [file ijms-24-00929-s001.zip › Standard curve, 200 a╠g Calibration results of standard samples.pdf]

Calibration：

ID#:4 Mass:TIC Name:Trehalose Derivative

f(x)=282765.584992*x+6617975.540630

rr2=0.993888

Area
5609148.00
10396488.00
17746241.00
30496807.00
51834063.00

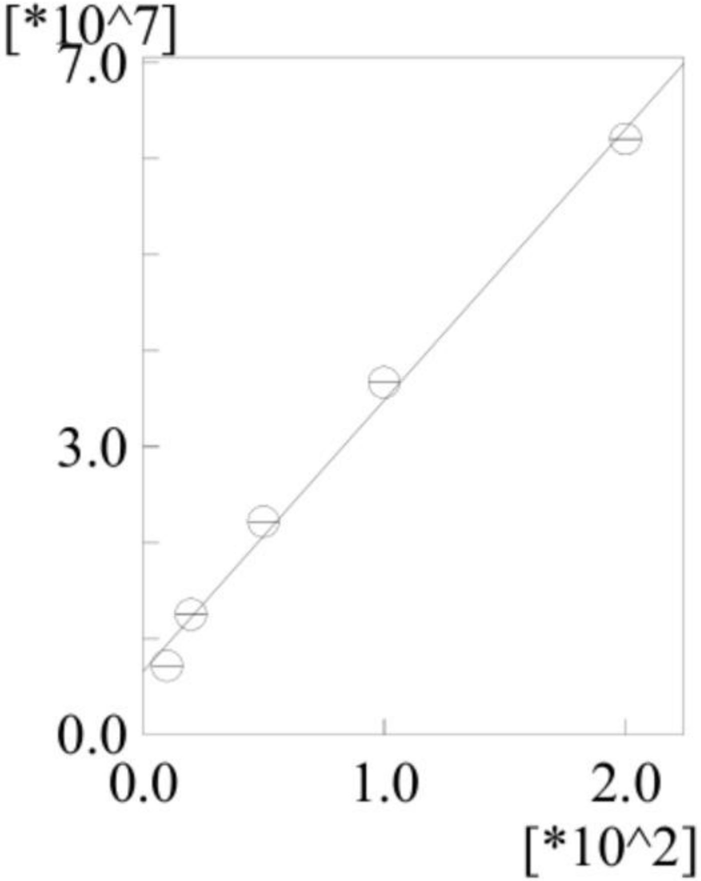

#	Conc. (μg)	Area
3	10.000	7152305.00
4	20.000	12530608.00
5	50.000	22152542.00
6	100.000	36694246.00
7	200.000	62011099.00

STD-200μg：

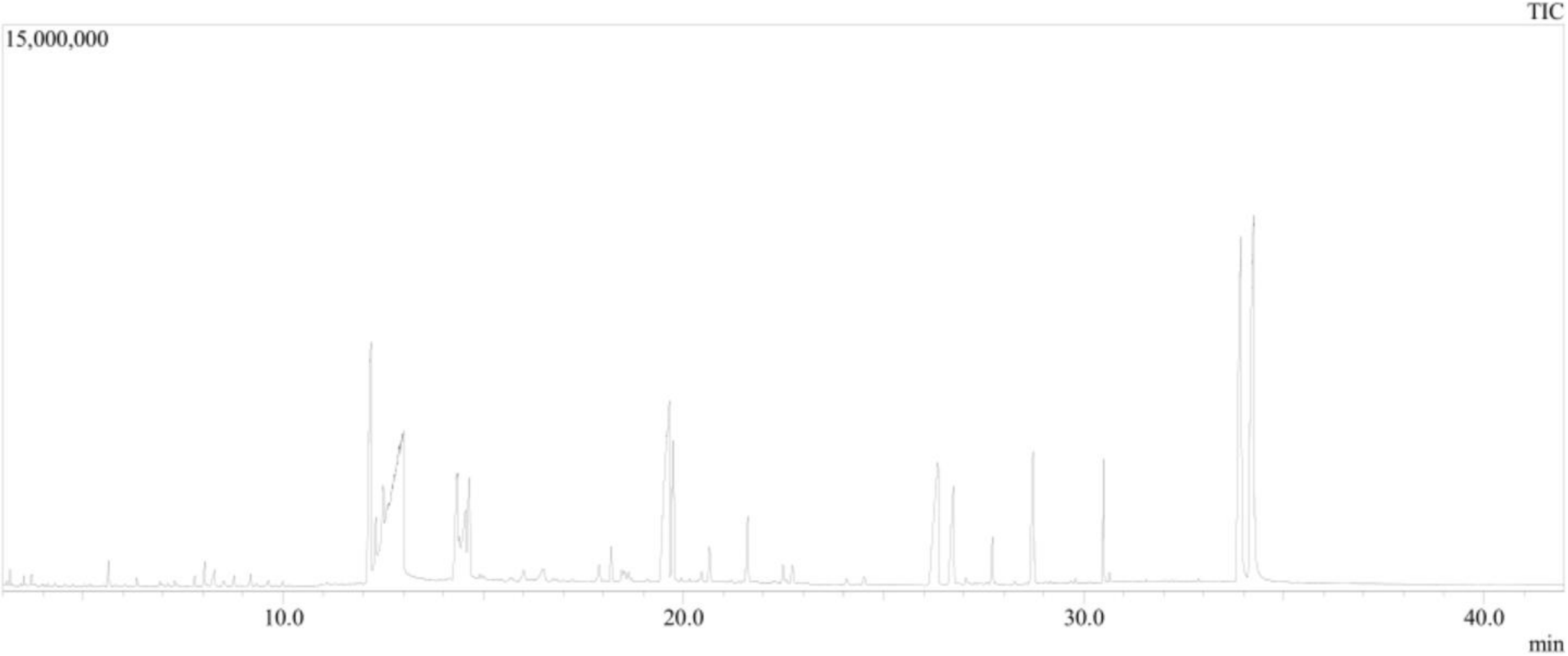

Quantitative Result Table

ID#	Name	R.Time	m/z	Area	Height	Conc.	Conc.Ur
	Trehalose Derivative	34.247	TIC	62011099	9746636	195.898	µg
