# Supplementary material for: Genome-Wide Identification and Characterization of the Trehalose-6-Phosphate Synthetase Gene Family in Chinese Cabbage (Brassica rapa) and Plasmodiophora brassicae during Their Interaction
Source: Int J Mol Sci. 2023 Jan 4;24(2):929. doi: 10.3390/ijms24020929 (PMC9864397; doi:10.3390/ijms24020929)
Supplement: Supplementary file 1 [file ijms-24-00929-s001.zip › Supplementary Table S1.pdf]

Supplementary Table S1. The qRT-PCR primer for *BrTPS* and *PbTPS*

Gene	primer
BrTPS1a-F	TCTGGTGCCATGATTACC
BrTPS1a-R	AGCCCAAGTATCCGAGT
BrTPS1b-F	CAGTTCGTATCACATCCCTCTC
BrTPS1b-R	GAACTGCCAACAGCTTCATTAG
BrTPS2-F	TGACCACACCAGTTGATTATGT
BrTPS2-R	ACTTGGATGACACGACTTCTG
BrTPS4-F	CTCGACACGATCAAAGGGATAC
BrTPS4-R	GCACTGCAATCTGAAGTAACAAA
BrTPS5a-F	GCCATTCTTGTGGATTAT
BrTPS5a-R	CCATTCGGTTAGTGTCTT
BrTPS5b-F	AAGACGCTAACCGAATGG
BrTPS5b-R	CATCACTGGCTCTGCTATTT
BrTPS6-F	CTGAACGCTGACTTGATTG
BrTPS6-R	TGACCCATGTGGATACCA
BrTPS7a-F	CGATCAAGCTTCCTCCGATAAT
BrTPS7a-R	GTCGTTTCGTCTCTCGGATTT
BrTPS7b-F	CTACGCTCGTCACTTCCTAAC
BrTPS7b-R	TACCCACAGTCCTACCGTAATA
BrTPS8-F	CAGGCAAGGAGGGAGTC
BrTPS8-R	TCTTCGCCCAGTAACCA
BrTPS9-F	CTTGGGACTTGCTGGAC
BrTPS9-R	AGTGAGCCTATGTAAACGAAC
BrTPS10a-F	GGGTTTAGGCTCATCGC
BrTPS10a-R	TTCAACGCAGACATCACA
BrTPS10b-F	GATTACGCACGGCATTT
BrTPS10b-R	CCTTCTTACCACGATACTTTT
BrTPS11-F	TGGATTACGATGGGACG
BrTPS11-R	AGACCGAGATTCGCACA
18SrRNA-F	GTTCTTAGTTGGTGGAGCGATTT
18SrRNA-R	ACCTGTTATTGCCTCAAACTTCC
PbTPS1-F	GCTATGCGATGCTGTCTTG
PbTPS1-R	GGTAATGCCACGAGATGAAC
PbTPS2-F	AGGGAGTCTACATCAGGTGG
PbTPS2-R	GGTCGTATCGGACAACATC
PbTPS3-F	TTTCTCGTGGTTCCCTGAG

PbTPS3-R	CCATTGACCCTTTCGTGAC
PbTPP-F	ACCATTGGCATCTTCCTG
PbTPP-R	CCATCTGAACGAAATCGC
PbActin-F	GGGACATCACCGACTACCTG
PbActin-R	ACTGCTCCGAGTTGGACATC
