# Supplementary figures and images for: Genome-Wide Identification and Characterization of the Trehalose-6-Phosphate Synthetase Gene Family in Chinese Cabbage (Brassica rapa) and Plasmodiophora brassicae during Their Interaction
Source: Int J Mol Sci. 2023 Jan 4;24(2):929. doi: 10.3390/ijms24020929 (PMC9864397; doi:10.3390/ijms24020929)

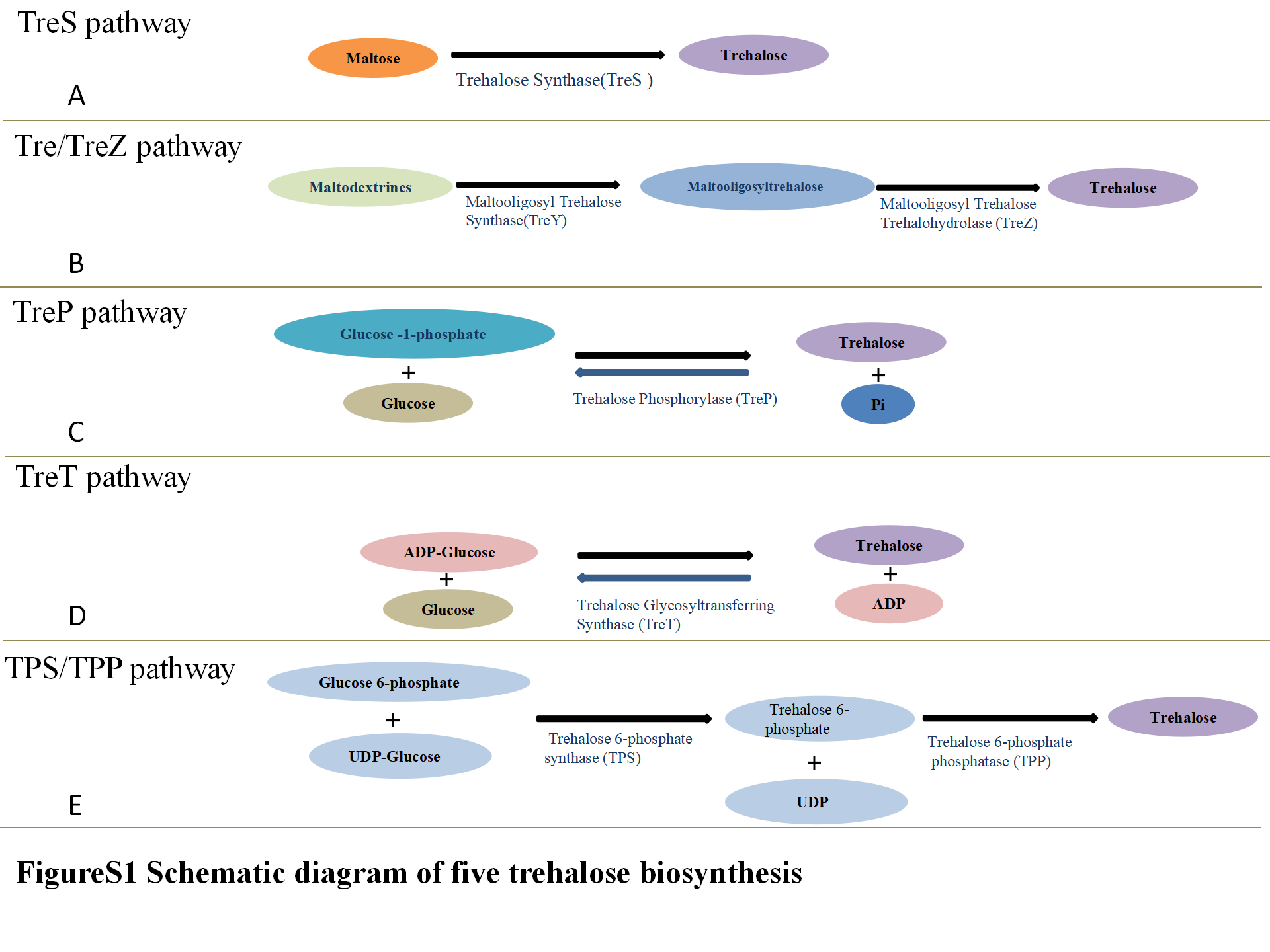

Supplement: Supplementary file 1 [file ijms-24-00929-s001.zip › Figure S1.tif]
